# Cellular Therapy Updates in B-Cell Lymphoma: The State of the CAR-T

**DOI:** 10.3390/cancers13205181

**Published:** 2021-10-15

**Authors:** Zachary D. Crees, Armin Ghobadi

**Affiliations:** Department of Medicine, Division of Oncology, Washington University School of Medicine, St. Louis, MO 63108, USA; arminghobadi@wustl.edu

**Keywords:** CAR-T cell, adoptive cell therapy, chimeric antigen receptor, B-cell lymphoma, tisagenlecleucel, axicabtagene ciloleucel, brexucabtagene autoleucel, lisocabtagene maraleucel, cytokine release syndrome, immune effector cell-associated neurotoxicity syndrome

## Abstract

**Simple Summary:**

B-cell lymphomas are the most commonly occurring blood cancer and the second leading cause of cancer-related death among blood cancers. Chemotherapy and stem cell transplantation have long served as the standard therapies for relapsed or refractory aggressive B-cell lymphomas with very poor survival, historically. Recently, the development of multiple chimeric antigen receptor T-cell (CAR-T) products has translated into dramatically improved outcomes and survival for patients with relapsed or refractory B-cell lymphoma. Meanwhile, basic, translational and clinical development within the field has progressed rapidly. The aim of this review is to summarize the current state of the art of CAR-T therapies for B-cell lymphomas within this rapidly evolving field, focusing on current United States Food and Drug Administration (US FDA)-approved products and a selection of promising areas of future clinical development.

**Abstract:**

Non-Hodgkin Lymphoma accounts for >460,000 cases and >240,000 deaths globally and >77,000 cases and >20,000 deaths in the U.S. annually, with ~85% of cases being B-cell malignancies. Until recently, patients with relapsed/refractory B-cell lymphoma following standard chemotherapy in combination with anti-CD20 monoclonal antibodies and autologous stem cell transplantation experienced a median overall survival (OS) of <6 months. However, with the approval of four different CD-19 CAR-T therapies between 2017 and 2021, approximately 60–80% of patients receiving CAR-T therapy now achieve an objective response with >3 years median OS. Here, we review the current state of the art of CD19 CAR-T therapies for B-cell lymphomas, focusing on current updates in US FDA-approved products, along with their associated efficacy and toxicities. Lastly, we highlight a selection of promising clinical developments in the field, including various novel strategies to increase CAR-T therapy efficacy while mitigating toxicity.

## 1. Introduction

Non-Hodgkin Lymphoma (NHL) is the most common hematologic neoplasm, representing a relatively heterogenous group of lymphoproliferative malignancies arising from lymphoid tissues [1,2,3,4,5,6,7,8]. NHL can be broadly categorized into T-cell and B-cell lymphomas, with B-cell lymphomas comprising ~85% of all NHL [2]. B-cell NHL can be further sub-classified, ranging from relatively indolent lymphomas to highly aggressive lymphomas [9,10].

Diffuse large B-cell lymphoma (DLBCL) represents the most frequently occurring aggressive B-cell lymphoma. Standard therapy for advanced DLBCL involves multi-agent chemoimmunotherapy, typically with rituximab, cyclophosphamide, doxorubicin, vincristine and prednisone (R-CHOP), which is associated with long-term remissions in 75–80% of patients [11]. However, depending on the International Prognostic Index (IPI) and the presence of adverse genetic features (*MYC, BCL2, BCL6*), as many as 50% of patients will be refractory to R-CHOP or will experience disease relapse after an initial response [12]. In such patients with high-risk disease, higher-intensity chemoimmunotherapy regimens may be utilized in an effort to reduce the rate of relapse. However, for those who develop relapsed or refractory (R/R) disease, <40% will achieve a durable response to salvage chemotherapy [13,14]. Meanwhile, among those who receive autologous stem cell transplantation (ASCT), up to 50% will ultimately relapse post-ASCT [15,16]. Similar to DLBCL, high-grade (3B) follicular lymphoma (FL), primary mediastinal large B-cell lymphoma (PMBCL), high-grade B-cell lymphoma (HGBCL) not otherwise specified (NOS) and transformed FL (tFL) represent related lymphoma subtypes that are generally treated with combination chemoimmunotherapy followed by salvage chemotherapy and/or ASCT for R/R disease with relatively similar efficacy [17].

Mantle cell lymphoma (MCL) represents a unique subtype of aggressive NHL, which tends to present in older patients and with a more indolent presentation at diagnosis compared to other aggressive lymphomas. Nevertheless, MCL is generally thought to be incurable, with a median OS ranging broadly from 5–10 years depending on multiple factors including disease risk and treatment response [18,19]. Advanced age (>70 years), elevated LDH, high WBC, complex karyotype, increased Ki-67, high mitotic rate, elevated beta-2 microglobulin and the presence of TP53 mutations have all been described as adverse risk factors associated with poor prognosis and decreased responses to therapy [19,20,21,22]. While there is no single standard induction regimen for MCL, commonly used multi-agent chemoimmunotherapy regimens may include, but are not limited to, bendamustine plus R (BR), R-CHOP with R maintenance or R-CHOP with consolidation ASCT [23,24,25]. In certain subgroups, higher-intensity induction may be considered using regimens such as rituximab, hyperfractionated cyclophosphamide, vincristine, doxorubicin and dexamethasone, alternating with high-dose methotrexate and cytarabine (R-Hyper-CVAD/MTX/Ara-C) or R-CHOP alternating with rituximab, dexamethasone, cytarabine and a platinum chemotherapy (R-DHAP) or rituximab and high-dose cytarabine [23,24,25]. Nevertheless, the majority of patients will experience R/R disease with no standard salvage regimen and poor long-term outcomes often characterized by serial relapse.

The concept of immune surveillance of neoplastic tissues was proposed in the early 1900s by Paul Ehrlich alongside his seminal studies of humoral immunity [26]. Over the following decades, numerous scientific advances in the understanding of humoral and cell-mediated immune effector functions coupled with technological advances have enabled the development of cellular-based cancer therapeutics. One critical advance in the development of cellular immunotherapy was that of Jim Allison and colleagues, who first described the T-cell receptor in 1983 [27]. Subsequent research further elucidated the structure and function of the TCR, including the highly variably TCR-alpha and -beta chains, which play a pivotal role in recognizing antigens in the context of major histocompatibility complex (MHC); the -gamma, -delta and -epsilon chains; and the intracellular -zeta chains, which play a critical role in transmitting TCR ligand binding into intracellular T-cell signaling and activation [28]. Of note, TCR recognition of cancer neo-antigens and the subsequent regulation of T-cell effector functions targeting neoplastic cells represent a highly relevant aspect of immune system cancer surveillance. In 1988, Steven Rosenberg and collaborators published the first-in-human data using adoptive cell therapy (ACT) with tumor infiltrating lymphocytes (TILs) to treat metastatic melanoma with >50% response rates [29]. Rosenberg and colleagues quickly followed up by publishing data in 1990 on the use of ACT with retroviral gene-transduced TILs to track the location and persistence of TILs in vivo, serving as the first-in-human use of gene-edited cellular therapies for the treatment of cancer [30]. These pivotal studies established the feasibility and potential effectiveness of cellular therapy platforms in the treatment of cancer, while demonstrating the safety of genetically modified cellular therapies.

In parallel to these landmark experiments defining the TCR’s structure/function and evaluating ACT with TILs, in 1987 Yoshihisa Kuwana, Yoshikazu Kurosawa and colleagues first published their work on the development of a genetically engineered T-cell expressing a 1st generation chimeric antigen receptor (CAR) containing the variable antigen-recognition domains of an antibody linked to the constant transmembrane and intracellular CD3-zeta signaling domains of a TCR [31]. By 1989, Gideon Gross and Zelig Eshhar published subsequent work describing the generation of a genetically engineered CAR T-cell (CAR-T) capable of recognizing antigens in the absence of MHC presentation and activating T-cell effector functions [32]. However, it would take nearly 20 years and numerous additional advances before these CAR-Ts successfully moved from the bench to the bedside, including increased transduction efficiency using both viral and non-viral methods, CAR-T activation and expansion and CAR construct optimization. In particular, the 1st generation CAR construct underwent notable revisions leading to a 2nd generation CAR, which contains the addition of either a CD28 or 41BB intracellular co-stimulatory domain to augment CD3-zeta mediated intracellular signaling and optimize T-cell activation. In 2017, data from the pivotal ZUMA-1 trial evaluating the 2nd generation CD19 CAR-T therapy axicabtagene ciloleucel demonstrated the remarkable efficacy of CD19 CAR-T therapies and led to the first US FDA approval of a CAR-T therapy for the indication of R/R large B-cell lymphoma (LBCL) [4]. Since that time, additional pivotal trials evaluating 2nd generation CD-19 CAR-T products for B-cell lymphoma have led to three more FDA approvals, including tisagenlecleucel in 2018 [5], brexucabtagene autoleucel in 2020 [6] and lisocabtagene maraleucel in 2021 [7].

However, despite the notable efficacy and high remission rates observed in these pivotal studies, CD19 CAR-T therapies are also associated with unique and potentially life-threatening toxicities including cytokine release syndrome (CRS) and immune effector cell-associated neurotoxicity syndrome (ICANS). CRS is associated with elevated serum levels of pro-inflammatory cytokines, including interferon gamma (IFN-gamma), TNF, IL-6 and IL-10, which contribute to a systemic hyper-inflammatory syndrome characterized most commonly by fevers, hypotension and hypoxemic respiratory failure [33,34]. The pathophysiologic mechanism of ICANS is less well understood but may be related to underlying endothelial dysfunction and the leakage of elevated serum cytokine levels across the blood–brain barrier, resulting in an inflammatory encephalopathy [35]. Nevertheless, both immune effector cell (IEC)-associated toxicities present unique challenges for both clinical management and research development.

Here, we review the current state of the art of CD19 CAR-T therapies for B-cell lymphomas, focusing on current updates in US FDA-approved products, along with their associated efficacy and toxicities. Lastly, we highlight areas of promising clinical development including various novel strategies to increase CAR-T therapy efficacy, overcome post-CAR-T relapse and mitigate IEC-associated toxicity.

## 2. US FDA-Approved CAR-T Therapies for B-Cell Lymphomas

The details of US FDA-Approved CAR-T therapies for B-cell lymphomas can be seen in Table 1.

### 2.1. Axicabtagene Ciloleucel

Axicabtagene ciloleucel (axi-cel) (Yescarta) is a CD19-directed genetically modified autologous CAR-T immunotherapy that was approved by the US FDA in 2017 for the treatment of adult patients (≥18 years) with R/R LBCL after two or more lines of systemic therapy, including DLBCL NOS, DLBCL arising from FL, HGBCL and PMBCL. Additionally, axi-cel was recently granted FDA approval for use in FL relapsing after two or more lines of systemic therapy in 2021.

#### 2.1.1. CAR Construct

The axi-cel CAR consists of a 2nd generation CD19-directed extracellular domain linked via a hinge and transmembrane domain to the intracellular CD28/CD3-zeta co-stimulatory domain transduced into unselected T-cells using a gamma-retrovirus vector.

#### 2.1.2. Efficacy

The efficacy of axi-cel was demonstrated in the pivotal ZUMA-1 trial, initially published in 2017 [4] with longer-term follow-up data in 2019 [36]. In the ZUMA-1 trial, patients on study did not receive bridging chemotherapy. Lymphodepleting chemotherapy (LDC) consisted of fludarabine (Flu) 30 mg/m^2^ and cyclophosphamide (Cy) 500 mg/m^2^ on days-5, -4 and -3 followed by infusion of axi-cel on day 0, which was dosed at 2.0 × 10^6^ CAR-T cells/kg (if >100 kg, maximum upper limit dose of 2.0 × 10^8^ CART cells/kg). A total of 111 adult patients with R/R DLBCL, PMBCL and tFL were enrolled, with 101 patients ultimately receiving axi-cel infusion and 108 patients being included in the modified intent-to-treat analysis. In the initial efficacy reports, the objective response (OR) rate was 82% with a complete response (CR) rate of 54%. The observed median time to response was 1 month, while 22 out of 57 patients (39%) who achieved an initial partial response (PR) or stable disease (SD) subsequently achieved a CR, suggesting ongoing CAR-T activity for months following the initial infusion. Most responses were observed within 6 months. In addition, the level of CAR-T expansion within the first 28 days of infusion was strongly correlated with response rate and risk of severe ICANS, but not CRS. At a median follow-up of 15.4 months, 42% of patients remained in remission with 40% being in a CR. OS at 18 months was 52%. The relatively high response rates in previously treatment-refractory patients coupled with a high proportion of long-term remissions generated considerable optimism regarding the potential of CAR-T therapies for B-cell lymphomas and led to the FDA approval of axi-cel for the indications listed above.

Longer-term data from ZUMA-1 at a median follow-up of 27.1 months demonstrated that the median duration of response (DOR) for all patients was 11.1 months [36]. However, among those achieving a CR, the median DOR was not reached and 39% of patients maintained a sustained remission on long-term follow-up. The median overall OS was not reached, while the median progression-free survival (PFS) was 5.9 months. Following FDA approval, real-world data on the use of axi-cel have also demonstrated similar results as reported with ZUMA-1, including data from the US Lymphoma CAR-T Consortium on 298 patients with R/R DLBCL achieving an OR rate of 82% and CR rate of 64% at a median follow-up of 12.9 months [37]. In addition, results from the ZUMA-5 trial evaluating axi-cel in indolent NHL (FL and marginal zone lymphoma (MZL)) demonstrated an OR rate of 94% with 76% of patients maintaining a sustained remission at 17.5 months median follow-up [38]. These data led to an accelerated FDA approval for axi-cel for the indication of FL relapsing after two or more lines of systemic therapy.

#### 2.1.3. Toxicity

In the ZUMA-1 trial, grade 3 or higher adverse events (AEs) occurred in 95% of patients, with the most common high-grade AEs being cytopenias (neutropenia 78%, anemia 43%, thrombocytopenia 38%). CRS and ICANS of any grade occurred in 93% and 64% of patients, respectively. However, severe (grade 3 or higher) CRS and ICANS occurred in only 13% and 28%, respectively. Treatment-related mortality was rare, with non-relapse mortality (NRM) accounting for only 3 out of 44 deaths on study (6%). A total of 43% of patients experiencing CRS and/or ICANS received tocilizumab (an anti-IL-6 receptor monoclonal antibody), while 27% received glucocorticoids without any apparent impact on treatment efficacy. In the larger US Lymphoma CAR-T Consortium experience following FDA approval, grade 3 or higher CRS and ICANS with axi-cel occurred in 7% and 31% of patients, respectively, with NRM accounting for 4.4% of deaths [37].

### 2.2. Tisagenlecleucel

Tisagenlecleucel (tisa-cel) (Kymriah) is a CD19-directed genetically modified autologous CAR-T immunotherapy US FDA approved for the treatment of adult patients with R/R LBCL after two or more lines of systemic therapy, including DLBCL NOS, DLBCL arising from FL and HGBCL. Of note, tisa-cel was also FDA approved for the treatment of pediatric and young adult (<26 years) patients with R/R B-cell acute lymphoblastic leukemia in 2017. However, this review will focus on B-cell lymphomas specifically.

#### 2.2.1. CAR Construct

The tisa-cel CAR consists of a 2nd generation CD19-directed extracellular domain linked via a hinge and transmembrane domain to the intracellular 41BB/CD3-zeta co-stimulatory domain transduced into selected T-cells using a lentivirus vector.

#### 2.2.2. Efficacy

The efficacy of tisa-cel for the treatment of LBCL was demonstrated in the pivotal JULIET trial initially published in 2019 [5]. In the JULIET trial, bridging chemotherapy was allowed, with 92% of patients receiving some form of bridging therapy during the manufacturing period. The most common agents used for bridging on study included some combination of the following agents: rituximab (54%), gemcitabine (40%), etoposide (26%), dexamethasone (25%), cisplatin (19%) and cytarabine (19%). LDC consisted of either Flu 25 mg/m^2^ and Cy 250 mg/m^2^ on days -5, -4 and -3 or bendamustine 90 mg/m^2^ for 2 days administered 1 week prior to infusion of tisa-cel on day 0, which was dosed at a median of 3.0 × 10^8^ CAR-T cells (range, 0.1 × 10^8^ to 6.0 × 10^8^). A total of 165 adult patients were enrolled, with 111 patients ultimately receiving tisa-cel infusion and 93 patients being included in the initial efficacy analysis with at least 3 months of follow-up. In the initial efficacy reports, the OR rate was 52% with a CR rate of 40%. Interestingly, 79% of patients who achieved CR and 65% of patients achieving any response maintained that response at 12 months of follow-up, with the median DOR not reached at a follow-up of 18 months. Additionally, no increased response rate was seen in the JULIET study based on the expansion or persistence of tisa-cel in vivo. These data led to the FDA approval of tisa-cel for the indications listed above.

Longer-term data from the JULIET trial demonstrated that 60.4% of patients maintained their response at 30 months, with the median DOR still not reached at a median follow-up of 40.3 months [39]. The median overall OS was 11.1 months for all patients. However, for patients who achieved a response at 3 months, the median OS was not reached with 40 months follow-up, highlighting the durable nature of responses to tisa-cel when they were achieved. Most recently in 2021, the 5-year outcomes from the JULIET trial with a median follow-up of 60.7 months demonstrated an OR rate of 58% with CR rate of 46% and median DOR of 61.4 months in patients with DLBCL [40]. Meanwhile, for patients with FL, the OR rate was 79% with a CR rate of 71% and median DOR not reached [40]. Interestingly, when late relapses were observed (>1 year after initial response), loss of CD19 expression on tumor cells by flow cytometry was not detected, suggesting the mechanism of late relapse to tisa-cel may be unrelated to antigen dropout. Following FDA approval, additional real-world data on the use of tisa-cel have demonstrated similar results as reported in JULIET, including data from the Center for International Blood and Marrow Transplant Research (CIBMTR) with 155 patients with R/R NHL achieving an OR rate of 61.8% and CR rate of 39.5% at a median follow-up of 11.9 months [41].

#### 2.2.3. Toxicity

In the JULIET trial, grade 3 or higher AEs occurred in 85% of patients, with the most common AEs being cytopenias (neutropenia 34%, anemia 48%, thrombocytopenia 33%). CRS and ICANS of any grade occurred in 58% and 21% of patients, respectively. However, severe (grade 3 or higher) CRS and ICANS occurred in only 22% and 12%, respectively. Treatment-related mortality was not observed in the JULIET trial, with three deaths reported within the first 30 days after infusion being related to the progression of disease. A total of 14% of patients experiencing CRS and/or ICANS received tocilizumab, while 10% received glucocorticoids without any apparent impact on treatment efficacy. In the real-world CIBMTR experience following FDA approval, grade 3 or higher CRS and ICANS with tisa-cel occurred in 11.6% and 7.5% of patients, respectively [41].

### 2.3. Brexucabtagene Autoleucel

Brexucabtagene autoleucel (brexu-cel) (Tecartus) is a CD19-directed genetically modified autologous CAR-T immunotherapy with accelerated approval for the treatment of adult patients with R/R MCL.

#### 2.3.1. CAR Construct

The brexu-cel CAR consists of a 2nd generation CD19-directed extracellular domain linked via a hinge and transmembrane domain to the intracellular CD28/CD3-zeta co-stimulatory domain transduced into selected T-cells using a gamma-retrovirus vector. Notably, while the CAR construct and transduction method for brexu-cel are similar to axi-cel, one significant difference in the manufacturing of brexu-cel is the selection of T-cells in order to ensure the removal of any CD19-expressing malignant cells prior to transduction of the CAR [6].

#### 2.3.2. Efficacy

The efficacy of brexu-cel was demonstrated in the pivotal ZUMA-2 trial, initially published in 2020 [6]. In the Zuma-2 trial, bridging chemotherapy using glucocorticoids and/or BTK inhibition (ibrutinib or acalibrutinib) was allowed, with 37% of patients receiving some form of bridging therapy. LDC consisted of Flu 30 mg/m^2^ and Cy 500 mg/m^2^ on days -5, -4 and -3 followed by infusion of brexu-cel on day 0, which was dosed at 2.0 × 10^6^ CAR-T cells/kg (if >100 kg, maximum upper limit dose of 2.0 × 10^8^ CART cells/kg). A total of 74 adult patients with R/R MCL were enrolled, with 68 patients ultimately receiving brexu-cel infusion and 60 patients being included in primary efficacy analysis. In the initial efficacy reports, the best OR rate was 93% with a CR rate of 67%. However, based on the intention-to-treat analysis, the OR rate was 85% with a CR rate of 59%. The median time to response was 1 month (range 0.8–3.1 months) and median time to CR was 3 months. Moreover, among the 42 patients who achieved an initial PR or SD, 57% went on to develop a CR within a median of 2.2 months, suggesting ongoing anti-tumor activity for months following the initial CAR-T infusion. At a median follow-up of 12.3 months, 57% of patients remained in remission. OS at 12 months was 83% with PFS of 61%. The relatively high response rates in previously treatment-refractory MCL patients coupled with durable remission rates at 12 months led to an accelerated approval of brexu-cel by the FDA for the indications listed above. Continued approval of brexu-cel will be contingent upon the results of an ongoing confirmatory trial demonstrating similar clinical benefit (NCT04880434).

#### 2.3.3. Toxicity

In the ZUMA-2 trial, grade 3 or higher AEs occurred in 99% of patients, with the most common high-grade AEs being cytopenias (neutropenia 85%, anemia 50%, thrombocytopenia 51%). Notably, 26% of patients experienced prolonged cytopenias of grade 3 or higher for more than 90 days. CRS and ICANS of any grade occurred in 91% and 63% of patients, respectively. However, severe (grade 3 or higher) CRS and ICANS occurred in only 15% and 31%, respectively. Treatment-related mortality was rare, with NRM accounting for two deaths (3%) due to infectious complications. A total of 59% of patients experiencing CRS and/or ICANS received tocilizumab, while 38% received glucocorticoids without any apparent impact on treatment efficacy.

### 2.4. Lisocabtagene Maraleucel

Lisocabtagene maraleucel (liso-cel) (Breyanzi) is a CD19-directed genetically modified autologous CAR-T immunotherapy FDA approved for the treatment of adult patients with R/R LBCL after two or more lines of systemic therapy, including DLBCL NOS, DLBCL arising from indolent NHL, HGBCL and FL (grade 3B).

#### 2.4.1. CAR Construct

The liso-cel CAR consists of a 2nd generation CD19-directed extracellular domain linked via a hinge and transmembrane domain to the intracellular 41BB/CD3-zeta co-stimulatory domain transduced into selected T-cells (1:1 CD4:CD8 ratio) using a lentivirus vector.

#### 2.4.2. Efficacy

The efficacy of liso-cel in the treatment of LBCL was demonstrated in the pivotal TRANSCEND trial, initially published in 2020 [7]. In the TRANSCEND trial, bridging chemotherapy was allowed, with 59% of patients receiving some form of bridging therapy during the manufacturing period. The most common bridging regimens used in >10% of patients were a combination of rituximab, gemcitabine and oxaliplatin (R-GEMOX) (11%), dexamethasone (11%) and radiotherapy. A number of other agents were used less frequently. LDC consisted of Flu 30 mg/m^2^ and Cy 300 mg/m^2^ for 3 days administered between 2 and 7 days prior to liso-cel infusion. Liso-cel was infused at three different dose levels in the TRANSCEND trial, including 50 × 10^6^ CAR-T cells, 100 × 10^6^ CAR-T cells and 150 × 10^6^ CAR-T cells, which were administered as sequential infusions of the two separate components of CD4+ and CD8+ CAR-T cells. There were no significant differences in efficacy or safety across the dose levels and therefore the recommended target dose level was determined to be ~100 × 10^6^ CAR-T cells. A total of 344 adult patients were enrolled, with 269 patients ultimately receiving at least one infusion of liso-cel and 256 patients being included in the efficacy analysis. The OR rate was 73% with a CR rate of 53% at a median follow-up of 18.8 months. Meanwhile, in the intention-to-treat analysis, the OR rate was 61% with a CR rate of 44%. The median duration of response was not reached at 12 months, with 55% of all patients and 65% of those in a CR maintaining remission. Median PFS was 6.8 months and OS was 21.1 months. These data led to the FDA approval of liso-cel for the indications listed above.

#### 2.4.3. Toxicity

In the TRANSCEND trial, grade 3 or higher AEs occurred in 79% of patients, with the most common high-grade AEs being cytopenias (neutropenia 60%, anemia 37%, thrombocytopenia 27%). Notably, CRS and ICANS were particularly rare relative to other FDA-approved CAR-T cell products, with CRS and ICANS of any grade occurring in 42% and 30% of patients, respectively. Moreover, severe (grade 3 or higher) CRS and ICANS occurred in only 2% and 10%, respectively. This translated into lower requirements for tocilizumab and glucocorticoids, with total of 20% of patients requiring tocilizumab and/or glucocorticoids. However, a total of seven (3%) patients did experience NRM with one case of each of the following: diffuse alveolar damage, pulmonary hemorrhage, multiple organ dysfunction syndrome, cardiomyopathy, leukoencephalopathy, septic shock and progressive multifocal leukoencephalopathy.

## 3. Promising Cellular Therapy Research for B-Cell Lymphomas

The results of the presented pivotal trials and subsequent FDA approval of four different CD19-directed CAR-T products for the treatment of a spectrum of R/R B-cell lymphomas have dramatically improved patient outcomes [4,5,6,7]. However, refractoriness to CAR-T treatment, relapse after initial response, CAR-T toxicity and the cost of CAR-T therapies represent significant areas for improvement upon the current standard of care. Here, we present a selected review of promising translational and clinical research focused on addressing some of these unmet needs (Table 2).

### 3.1. Targeting New Antigens

Currently, the four different CAR-T therapies US FDA approved for B-cell lymphomas all target CD19, a commonly expressed mature B-cell antigen. However, the antigen recognition portion of the CAR consists of the single-chain variable (scFv) region of an antibody, allowing the antigen specificity of a CAR to be customizable and the development of CARs targeting a broad array of antigens. In addition, the selection of antigens that are highly and consistently expressed on malignant cells while not expressed on normal, healthy tissues is an important consideration in optimizing on-target on-tumor CAR-T activity (i.e., cancer-cell killing) while minimizing on-target off-tumor activity (i.e., toxicity to normal tissue). Meanwhile, antigen dropout with loss of CD19 on malignant cells represents one well-described mechanism of relapse after initial responses to CD19 CAR-T treatment. Therefore, the development of CAR-Ts to target alternative antigens remains an active area of investigation.

Following CD19, targeting the antigens CD22 and CD20 represents two of the more promising strategies in clinical development for CAR-T therapies for B-cell lymphoma. CD20 is a commonly expressed mature B-cell antigen and is also expressed on a large proportion of B-cell lymphomas, whereas CD22 is an inhibitory co-receptor expressed on a range of mature B-cells, immature B-cells and B-cell lymphomas. As of 2021, as many as 39 different CD22 CAR-T products and 26 CD20 CAR-T products have been reported to be in various stages of pre-clinical and clinical development, including 10 phase II clinical trials of CD22 CAR-Ts and 8 phase II trials for CD20 CAR-Ts underway [42]. Meanwhile, other antigen targets for B-cell lymphomas have been explored in pre-clinical development, including B-cell activating factor receptor (BAFF-R), CD79a, CD37 and Ig-kappa, as well as others. The BAFF-R is a pro-survival receptor expressed on B-cells and a large proportion of malignant B-cells that plays an important role in the proliferation of both normal B-cells and malignant lymphoma in response to BAFF, a member of the tumor necrosis factor (TNF) family of ligands [43]. Preclinical mouse models have shown BAFF-R CAR-T cells are capable of killing B-cell lymphoma cells, and notably remain efficacious against malignant B-cells that have lost CD19 antigen expression [44]. CD79a is a cell surface protein that forms a dimer with the immunoglobulin of a B-cell to form the B-cell antigen receptor. Targeting CD79a with cellular therapies has been explored in pre-clinical studies with signs of efficacy [45]. Mature B-cells also commonly express immunoglobulins (Ig) with non-restricted (i.e., polyclonal) kappa and lambda expression, whereas malignant B-cells commonly are monoclonal and thus express a restricted Ig-kappa or lambda phenotype. CAR-Ts targeting Ig-kappa have been explored in pre-clinical and early-phase clinical trials with minimal toxicity and the notable benefit of sparing the lambda-expressing lymphocytes [46]. CD37 is an antigen that is widely expressed on various immune cells, but is found in highest abundance in B-cells and a large number of lymphomas. CD37 CAR-Ts have been explored in preclinical models with signs of activity, but the potential for on-target off-tumor toxicity to CD37+ immune cells remains an important consideration [47]. In summary, the development of CAR-Ts targeting new malignancy-associated antigens is underway in multiple clinical trials and is likely to yield new therapeutic options for patients with antigen-positive B-cell malignancies.

### 3.2. Multi-Specific CARs

Antigen dropout, as previously noted, is one mechanism of relapse after CD19 CAR-T therapies. In addition, B-cell lymphomas can be heterogenous and dynamic in their antigen presentation. Therefore, one strategy to increase the effectiveness of CAR-T cells has been the development of bi-specific and/or multi-specific CAR-T cells that express CARs with affinity to more than one antigen. Both CD19/CD20 and CD19/CD22 bi-specific CAR-Ts have been evaluated in early-phase clinical trials with acceptable safety profiles and signals of efficacy [48,49]. Various other tri-specific and novel multi-specific strategies have been reported in pre-clinical stages of development [50]. One strategy involves the co-administration of a mixture of two or more CAR-T lines with each CAR-T expressing a different CAR construct with single-antigen specificity. Another strategy described involves the generation of a bi-cistronic CAR-T line with each CAR-T expressing two different CAR constructs that each have single-antigen specificity. Lastly, the generation of tandem CAR-Ts has been described, with the infusion of a single CAR-T line that expresses a single CAR construct that has an extracellular domain with scFv portions in a number of potential configurations capable of recognizing two or more antigens [51,52].

### 3.3. Allogeneic “Off the Shelf” CAR-Ts

**Figure 1:**
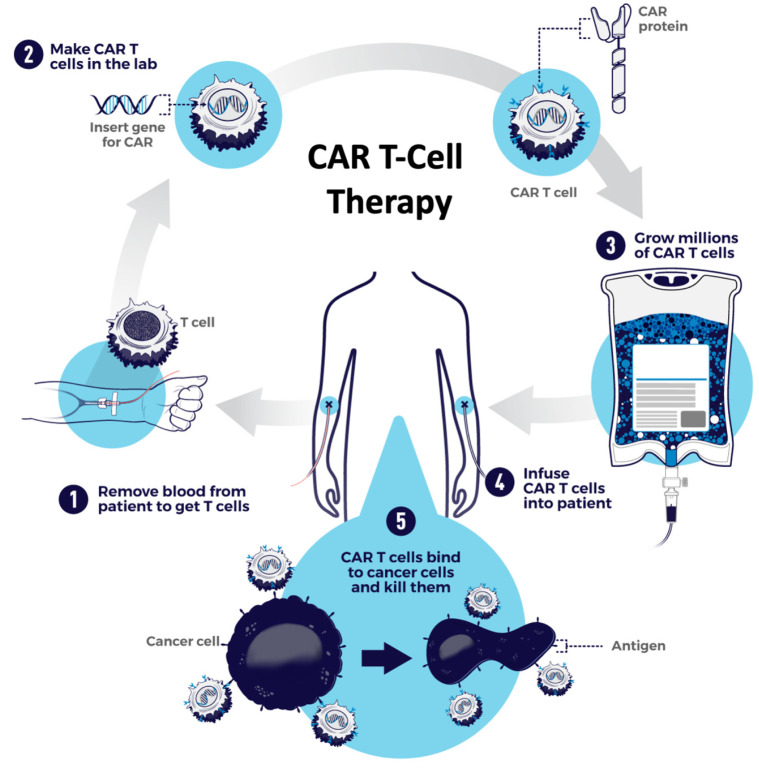
The general manufacturing process of CAR-T cells, involving (1) leukapheresis, (2) transportation of T-cells to a GMP facility where they are genetically modified and transduced with the CAR, (3) are expanded, purified and undergo quality checks, (4) then are transported back to the treating facility where the patient is treated with lymphodepleting chemotherapy and then infused with the CAR-Ts, (5) which then engage with cancer cells by recognition of the target antigen to mediate the therapeutic effect. Credit: NIH-NCI, open source image.

One significant limitation to the use of CAR-Ts is the considerable time and financial resources necessary for the manufacturing process. CAR-T manufacturing typically takes a minimum of 3–6 weeks and involves patients undergoing leukapheresis to collect autologous T-cells, the transport of the T-cells to a GMP facility, the isolation/selection/stimulation of the necessary T-cells, gene editing (depending on the CAR-T product), CAR transduction, expansion/purification of CAR-positive T-cells, quality control checks, the transportation of cells back to the site of patient care, the administration of lymphodepleting conditioning chemotherapy and finally the infusion of the CAR-T cells into the patient (Figure 1). During this process, the manufacturing time, potential manufacturing failures/delays and risk of disease progression in patients can all represent significant barriers to treatment. In addition, the logistics and personalized generation of each CAR-T treatment significantly increase costs. Therefore, considerable interest and research has focused on the concept of developing “off-the-shelf” CAR-T products that can be manufactured and stored ahead of time and are available to be administered to a patient in need within a matter of days, rather than multiple weeks.

One of the most promising approaches to addressing these challenges with autologous CAR-Ts is the development of allogeneic CAR-T cells, which could make use of T-cells collected from healthy donors manufactured into CAR-Ts in advance, stored in cryopreservation and then shipped to the treating facility on demand. Such an approach would significantly minimize time to treatment, eliminate manufacturing delays/failures and would also allow for significant reductions in cost. However, a major barrier to the use of allogeneic CAR-Ts has been the risk of T-cell-mediated graft-versus-host disease (GVHD). Fortunately, advances in the field of gene editing using various techniques, such as zinc finger nucleases, transcription activator-like effector nucleases (TALENs) and CRISPR/Cas-9 gene editing, have enabled the potential to modify allogeneic T-cells during the manufacturing process by editing genomic sequences in the constant regions of the endogenous alpha- or beta-subunits of the TCR and the HLA-A locus to disrupt allo-antigen recognition and T-cell activation [53,54,55,56]. The resulting CAR-Ts have been termed “universal” CAR-Ts or UCARTs, owing to their ability to be universally administered to HLA-unmatched recipients with limited risk of allo-reactivity and GVHD. UCARTs have been explored in preclinical studies with promising pre-clinical activity targeting a variety of antigens [56,57,58,59]. Currently, multiple clinical trials are underway to determine the efficacy and toxicity of various allogeneic CAR-T products, with the potential to significantly improve upon the manufacturing challenges and costs associated with autologous CAR-T therapies.

### 3.4. Optimizing CAR-T Signaling, Expansion and Persistence

**Figure 2:**
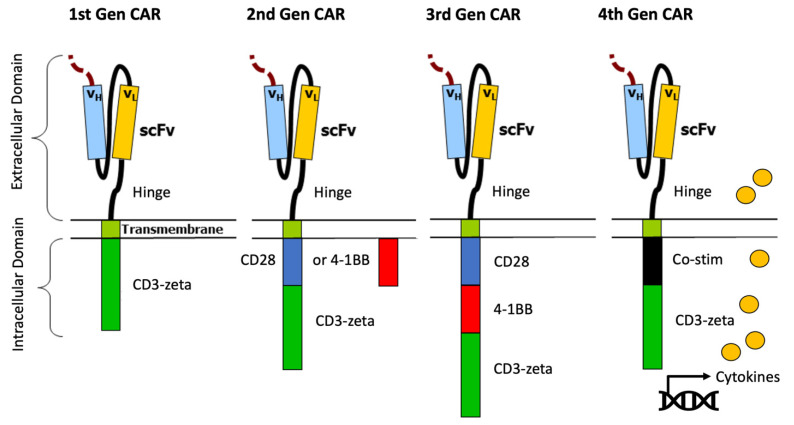
The 1st generation CAR construct includes an extracellular domain with a single-chain variable fragment (scFv) antigen recognition region connected via a hinge and transmembrane domain to the CD3-zeta portion of a TCR. Of note, the scFv is a chimeric protein that includes the variable heavy (VH) and variable light (VL) chains of an immunoglobulin connected via a linker peptide. Second generation CARs include the addition of co-stimulatory domains (CD28 or 4-1BB) to the intracellular domain. Third generation CARs include the addition of a combination of multiple co-stimulatory domains (e.g., CD28 and 4-1BB) to the intracellular domain. Fourth generation CARs include a unique co-stimulatory domain that facilitates increased production of specific cytokines, which augment CAR-T activation and effector functions. Open source image, credit: Mxpule.

During early development of CAR-T cells, the first generation of CARs consisted of an extracellular antigen recognition (scFv) domain connected via a hinge and transmembrane domain to the intracellular domain, which consisted of the CD3-zeta portion of the TCR [31,32]. These early CAR-Ts demonstrated the proof of principle. However, it was not until the 2nd generation CAR-Ts with additional co-stimulatory endodomains (CD28 or 4-1BB) were developed that the true clinical efficacy of CAR-Ts was fully demonstrated [4,5,6,7]. Subsequent research has led to additional co-stimulatory domain modification in the form of 3rd generation CAR-Ts, for example, using CD28/4-1BB/CD3-zeta or CD28/OX-40/CD3-zeta [60]. Additionally, 4th generation CAR-Ts with additional endodomains have been described, which are capable of inducing the production and release of cytokines such as IL-12, IL-15 or IL-18 to enhance the activity of CAR-T cells, change the tumor microenvironment, recruit additional inflammatory cells and achieve CAR-T self-activation by autocrine pathways [61,62] (Figure 2). Additionally, the degree of CAR-T expansion and persistence has been linked to increased response rates in some but not all FDA-approved CAR-T products [4,5]. Therefore, ongoing research has sought to understand the underlying mechanisms of CAR-T expansion and to evaluate interventions that may increase CAR-T expansion and/or persistence. One such approach to improve in vivo CAR-T expansion being currently evaluated in an ongoing phase 1/2 clinical trial is the administration of a long-acting, humanized recombinant IL-7 receptor agonist in combination with CAR-T infusion, which has been shown to increase T-cell expansion and persistence in a number of other clinical trials for other diseases. Additionally, the up-regulation of programmed cell death protein 1 (PD-1) has been described as a marker of T-cell exhaustion, while the up-regulation of PD-L1 on malignant cells has been shown to inhibit CAR-T effector functions [63]. Clinical trials investigating the addition of check-point inhibitors to CAR-T are ongoing with early signs of clinical activity, but also with increased AE rates including ICANS [64,65,66]. Currently, there are multiple pre-clinical and clinical trials ongoing to evaluate a broad array of additional strategies to optimize CAR-T function and efficacy in both FDA-approved CAR-Ts as well as investigational CAR-Ts.

### 3.5. Reducing CAR-T Toxicity

The considerable effectiveness of CD19 CAR-T therapies for B-cell malignancies has dramatically improved clinical outcomes. However, these therapies are also associated with significant toxicities including CRS and ICANS. CRS has been associated with elevated serum levels of cytokines, such as IFN-gamma, TNF, IL-6 and IL-10, which contribute to a systemic hyper-inflammatory syndrome characterized by fevers, hypotension and, in severe cases, hypoxemic respiratory failure and multi-organ failure [33,34]. Overall, CRS rates across the four US FDA-approved CD19 CAR-T products for B-cell lymphoma range from 42–93%, with 2–22% being grade 3 or higher [4,5,6,7]. Meanwhile, the underlying mechanism of ICANS is incompletely characterized but is thought to be mediated in part by endothelial damage and/or activation leading to the leakage of elevated serum cytokine levels across the blood–brain barrier and an inflammatory encephalopathy [35]. ICANS rates across the four US FDA-approved CD19 CAR-T products for B-cell lymphoma range from 21–64%, with 10–31% being grade 3 or higher [4,5,6,7]. Interestingly, CAR-Ts with a CD28 costimulatory domain seem to be associated with higher rates of CRS and ICANS compared to CAR-Ts with 4-1BB costimulatory domains, although these various CAR-Ts have not been compared in head-to-head trials. Moreover, making cross-trial comparisons of CRS and ICANS rates between different CD19 CAR-T therapies for B-cell lymphomas has been further confounded by different methods used for grading CRS and ICANS. As a result of the need to standardize the grading and management of IEC-associated toxicities, the American Society of Transplantation and Cellular Therapy (ASTCT) developed and published a consensus guideline for grading and reporting CRS and ICANS [67].

**Figure 3:**
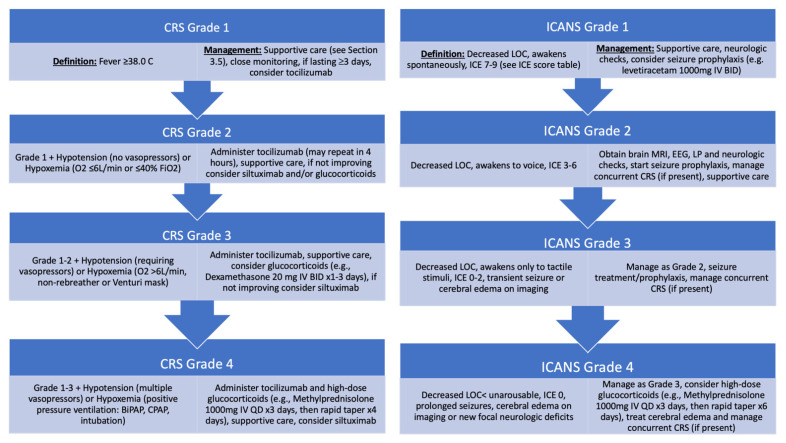

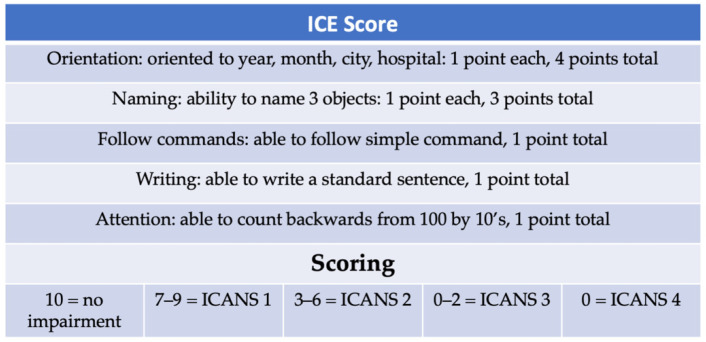
General guidance regarding management of CRS and ICANS, including ICE scoring table.

In the clinical management of CRS and ICANS, the use of tocilizumab and glucocorticoids along with supportive care has become the standard (Figure 3). In addition, supportive care must be adjusted to meet each patient’s medical needs in accordance with the respective grade of CRS and/or ICANS, including, but not limited to: close monitoring with continuous telemetry and pulse oximetry; IV fluids and vasopressors for hypotension; supplemental O2 with or without non-invasive or invasive positive pressure ventilatory support for hypoxemia and respiratory distress; G-CSF, thrombopoietin mimetics and transfusion support for prolonged cytopenias; a complete infectious work-up and prompt initiation of empiric broad spectrum antibiotics for neutropenic fever; and acetaminophen for pyrexia, IVIG for clinically significant immunoparesis due to prolonged hypogammaglobulinemia and appropriate antimicrobial prophylaxis for herpes simplex, *Pneumocystis*, fungal, bacterial and other opportunistic infections, as indicated [67]. Meanwhile, ongoing research efforts to reduce and/or prevent these IEC-associated toxicities have taken numerous forms. One such approach has focused on the modification of the CAR construct to mitigate CRS and ICANS, given that the CAR and its individual domains contribute significantly to downstream CAR-T activation and cytokine production. For example, a phase I study of a CD19 CAR-T with a modified CD8-alpha hinge/transmembrane domain demonstrated significantly lower levels of inflammatory serum cytokines, no ICANS and no grade 3 or higher CRS while still achieving >50% OR rates [68]. In some cases, on/off switches and “suicide” switches have been engineered into the CAR construct to allow physicians the ability to disable CAR-Ts when a patient experiences high-grade AEs [69]. In addition, a number of clinical trials are ongoing to evaluate the administration of novel prophylactic strategies to reduce the risk of CRS and ICANS while not hindering the therapeutic effect, including the inhibition of the JAK/STAT pathway (NCT04071366). Meanwhile, there are numerous other pre-clinical and clinical studies underway aimed at modeling and ultimately improving the toxicity profile of CAR-Ts.

## 4. Conclusions

B-cell lymphomas are the most commonly occurring hematologic malignancy and the second leading cause of death among hematologic neoplasms. Historically, outcomes for R/R B-cell lymphoma treated with traditional chemoimmunotherapy have been poor. The development and subsequent US FDA approval of four different CD19 CAR-T therapies for B-cell lymphomas have dramatically improved outcomes and survival for these patients. However, refractoriness to CAR-T therapy, post-CAR-T relapse, CAR-T toxicity and the financial costs associated with CAR-T therapy represent significant challenges to the field. Fortunately, a number of translational and clinical studies are underway with the aim of solving these unmet needs, including targeting new cancer antigens, generating effective/safe multi-specific and “off-the-shelf” allogeneic UCARTs, optimizing CAR-T function and reducing CAR-T-related medical and financial toxicity. Moreover, CAR-T therapy as a broad anti-cancer platform to treat a diverse array of hematologic and solid malignancies is rapidly growing with over 1150 CAR-T cell therapies in development globally in 2021, including 512 ongoing phase I–III human trials targeting numerous antigens for both hematologic (e.g., CD19, BCMA, CD22, CD20, CD123, CD33) and solid malignancies (HER2, PSMA, MSLN, GD2, EGFR, GPC2/3, NYESO1, etc.).

## Figures and Tables

**Table 1:** Summary table of the 4 FDA-approved CD19 CAR-T products for B-cell lymphomas and the results of their pivotal trials. Abbreviations: US: United States; FDA: Food and Drug Administration; gen: generation; DLBCL: diffuse large B-cell lymphoma; tFL: transformed follicular lymphoma; ALL: acute lymphoblastic leukemia; PMBCL: primary mediastinal B-cell lymphoma; MCL: mantle cell lymphoma; post-ASCT: post autologous stem cell transplantation; chemo: chemotherapy; Flu: fludarabine; Cy: cyclophosphamide; CAR-T: chimeric antigen T-cell; yrs: years; kg: kilogram; max: maximum; DL: dose level; OR: overall response; CR: complete response; Med.: median; DOR: duration of response; PFS: progression-free survival; OS: overall survival; mo: months; CRS: cytokine release syndrome; AE: adverse event.

	Axicabtagene Ciloleucel (axi-cel, Yescarta)	Tisagenlecleucel (tisa-cel, Kymriah)	Brexucabtagene Autoleucel (brexu-cel, Tecartus)	Lisocabtagene Maraleucel (liso-cel, Breyanzi)
US FDA Approval	2017: LBCL 2021: FL	2017: ALL (age < 26 years) 2018: LBCL	2020: MCL	2021: LBCL
CAR Construct	•2nd gen, CD28 •Retroviral vector	•2nd gen, 41BB •Lentiviral vector	•2nd gen, CD28 •Retroviral vector	•2nd gen, 41BB •Lentiviral vector
Pivotal Trial	ZUMA-1 [4] Phase 1/2	JULIET [5] Phase 2	ZUMA-2 [6] Phase 2	TRANSCEND [7] Phase 2
Study Population	111 enrolled; 101 dosed •76% DLBCL; 16% tFL; 8% PMBCL •79% refractory •21% post-ASCT	165 enrolled; 111 dosed •80% DLBCL; 18% tFL •54% refractory •49% post-ASCT	74 enrolled, 68 dosed •100% MCL •40% refractory •43% post-ASCT	344 enrolled, 269 dosed •51% DLBCL, 13% HGBCL, 6% PMBCL, 1% FL grade 3b •67% refractory •35% post-ASCT
Bridging Chemo	No	Yes, 59%	Yes, 37%	Yes, 59%
Lymphodepleting Chemo	Flu 30 mg/m^2^ and Cy 500 mg/m^2^ on Days −5, − 4, and − 3	•Flu 25 mg/m^2^ and Cy 250 mg/m^2^ on Days −5, − 4, and − 3 •Bendamustine (90 mg/m^2^) daily for 2 days	Flu 30 mg/m^2^ and Cy 500 mg/m^2^ on Days −5, − 4, and − 3	Flu 30 mg/m^2^ and Cy 300 mg/m^2^ × 3 days, 2–7 days before CAR infusion
CAR-T Dose	•2.0 × 10^6^ CAR-T cells/kg •If >100 kg, max. 2.0 × 10^8^ CAR-T cells	•Median, 3 × 10^8^ CAR-T cells •Range, 0.1–6.0 × 10^8^ cells	•2.0 × 10^6^ CAR-T cells/kg •If >100 kg, max. 2.0 × 10^8^ CAR-T cells)	•DL1: 50 × 10^6^ CAR-T cells (*n* = 45) •DL1: 100 × 10^6^ CAR-T cells (*n* = 183) •DL3: 150 × 10^6^ CAR-T cells (*n* = 41) (CD4:CD8 in 1:1 ratio)
Efficacy	OR: 82% CR: 54% Med. DOR: 11.1 mo Med. PFS: 5.9 mo OS at 18 mo: 52%	OR: 52% CR: 40% Med. DOR: NR at 17 mo Med. OS: 11.1 months	OR: 85% CR: 59% Med. DOR: NR at 12 mo PFS at 12 mo: 61% OS at 12 mo: 83%	OR: 61% CR: 44% Med. DOR: NR at 12 mo Med. PFS: 6.8 mo Med. OS: 21.1 mo
Safety	CRS: •All Grades: 93% •≥ Grade 3: 13% Neurotoxicity: •All Grades: 64% •≥ Grade 3: 28% Grade 5 AEs: 6%	CRS: •All Grades: 58% •≥ Grade 3: 22% Neurotoxicity: •All Grades: 21% •≥ Grade 3: 12% Grade 5 AEs: 3%	CRS: •All Grades: 91% •≥ Grade 3: 15% Neurotoxicity: •All Grades: 63% •≥ Grade 3: 31% Grade 5 AEs: 3%	CRS: •All Grade: 42% •≥ Grade 3: 2% Neurotoxicity: •All Grades: 30% •≥ Grade 3: 10% Grade 5 AEs: 0%

**Table 2:** Summary table of promising future directions of translational and clinical CAR-T development. Abbreviations: TALENs: transcription activator-like effector nucleases; CAR: chimeric antigen receptor; Gen: generation; PD-1: programmed cell death protein 1; PD-L1: programmed cell death protein ligand 1; JAK: janus kinase; STAT: signal transducer and activator of transcription.

Future Directions in Translational and Clinical CAR-T Development	
**Targeting New Antigens**	CD20, CD22, BAFF-R, CD29a, CD37, Ig-kappa and others
**Multi-specific CARs**	Co-administration of ≥2 antigen-specific CAR-Ts; Bi-cistronic CAR-Ts expressing ≥2 different antigen-specific CARs; Tandem CAR-Ts expressing 1 CAR with dual antigen specificity (furthest in clinical development: CD19/CD20 and CD19/CD22)
**Allogeneic “off-the-shelf” Universal CAR-Ts**	TCR and/or HLA modifications using various gene editing techniques (e.g., zinc finger nucleases, TALENs and CRISPR-Cas9)
**Optimizing CAR-T Function**	CAR modification (3rd and 4th Gen CAR-Ts), cytokine stimulation/priming (e.g., IL-7), checkpoint inhibition (e.g., PD-1/PD-L1)
**Reducing CAR-T Toxicity**	Prophylactic tocilizumab and/or glucocorticoids, CAR modification, CAR-T “on/off” switches, CAR-T “suicide” switches, targeted inhibitors of relevant inflammatory signaling pathways (e.g., JAK/STAT)

## References

[1] Fitzmaurice C., Akinyemiju T.F., Al Lami F.H., Alam T., Alizadeh-Navaei R., Allen C., Alsharif U., Alvis-Guzman N., Amini E., Global Burden of Disease Cancer Collaboration (2018). Global, Regional, and National Cancer Incidence, Mortality, Years of Life Lost, Years Lived with Disability, and Disability-Adjusted Life-Years for 29 Cancer Groups, 1990 to 2016: A Systematic Analysis for the Global Burden of Disease Study. JAMA Oncol..

[2] Siegel R.L., Miller K.D., Jemal A. (2020). Cancer statistics, 2020. CA Cancer J. Clin..

[3] Crump M., Neelapu S.S., Farooq U., Neste E.V.D., Kuruvilla J., Westin J., Link B.K., Hay A., Cerhan J.R., Zhu L. (2017). Outcomes in refractory diffuse large B-cell lymphoma: Results from the international SCHOLAR-1 study. Blood.

[4] Neelapu S.S., Locke F.L., Bartlett N.L., Lekakis L.J., Miklos D.B., Jacobson C.A., Braunschweig I., Oluwole O.O., Siddiqi T., Lin Y. (2017). Axicabtagene Ciloleucel CAR T-Cell Therapy in Refractory Large B-Cell Lymphoma. N. Engl. J. Med..

[5] Schuster S.J., Bishop M.R., Tam C.S., Waller E.K., Borchmann P., McGuirk J.P., Jäger U., Jaglowski S., Andreadis C., Westin J.R. (2019). Tisagenlecleucel in Adult Relapsed or Refractory Diffuse Large B-Cell Lymphoma. N. Engl. J. Med..

[6] Wang M., Munoz J., Goy A., Locke F.L., Jacobson C.A., Hill B.T., Timmerman J.M., Holmes H., Jaglowski S., Flinn I.W. (2020). KTE-X19 CAR T-Cell Therapy in Relapsed or Refractory Mantle-Cell Lymphoma. N. Engl. J. Med..

[7] Abramson J.S., Palomba M.L., Gordon L.I., Lunning M.A., Wang M., Arnason J., Mehta A., Purev E., Maloney D.G., Andreadis C. (2020). Lisocabtagene maraleucel for patients with relapsed or refractory large B-cell lymphomas (TRANSCEND NHL 001): A multicentre seamless design study. Lancet.

[8] Shankland K.R., Armitage J.O., Hancock B.W. (2012). Non-Hodgkin lymphoma. Lancet.

[9] Pileri S.A., Milani M., Orcioni G.F., Sabattini E. (1998). From the R.E.A.L. Classification to the upcoming WHO scheme: A step toward universal categorization of lymphoma entities?. Ann. Oncol..

[10] Harris N.L., Jaffe E.S., Diebold J., Flandrin G., Muller-Hermelink H.K., Vardiman J. (2000). Lymphoma classification – from controversy to consensus: The R.E.A.L. and WHO Classification of lymphoid neoplasms. Ann. Oncol..

[11] Bartlett N., Wilson W.H., Jung S.-H., Hsi E.D., Maurer M.J., Pederson L.D., Polley M.-Y.C., Pitcher B.N., Cheson B.D., Kahl B.S. (2019). Dose-Adjusted EPOCH-R Compared With R-CHOP as Frontline Therapy for Diffuse Large B-Cell Lymphoma: Clinical Outcomes of the Phase III Intergroup Trial Alliance/CALGB 50303. J. Clin. Oncol..

[12] Sehn L.H., Berry B., Chhanabhai M., Fitzgerald C., Gill K., Hoskins P., Klasa R., Savage K.J., Shenkier T., Sutherland J. (2007). The revised International Prognostic Index (R-IPI) is a better predictor of outcome than the standard IPI for patients with diffuse large B-cell lymphoma treated with R-CHOP. Blood.

[13] Gisselbrecht C., Glass B., Mounier N., Gill D.S., Linch D.C., Trneny M., Bosly A., Ketterer N., Shpilberg O., Hagberg H. (2010). Salvage Regimens With Autologous Transplantation for Relapsed Large B-Cell Lymphoma in the Rituximab Era. J. Clin. Oncol..

[14] Neste E.V.D., Schmitz N., Mounier N., Gill D., Linch D., Trneny M., Milpied N., Radford J., Ketterer N., Shpilberg O. (2016). Outcome of patients with relapsed diffuse large B-cell lymphoma who fail second-line salvage regimens in the International CORAL study. Bone Marrow Transplant..

[15] Nagle S.J., Woo K., Schuster S.J., Nasta S.D., Stadtmauer E., Mick R., Svoboda J. (2013). Outcomes of patients with relapsed/refractory diffuse large B-cell lymphoma with progression of lymphoma after autologous stem cell transplantation in the rituximab era. Am. J. Hematol..

[16] Hamadani M., Hari P., Zhang Y., Carreras J., Akpek G., Aljurf M.D., Ayala E., Bachanova V., Chen A.I., Chen Y.-B. (2014). Early Failure of Frontline Rituximab-Containing Chemo-immunotherapy in Diffuse Large B Cell Lymphoma Does Not Predict Futility of Autologous Hematopoietic Cell Transplantation. Biol. Blood Marrow Transplant..

[17] Radford J., Davies A., Cartron G., Morschhauser F., Salles G., Marcus R., Wenger M., Lei G., Wassner-Fritsch E., Vitolo U. (2013). Obinutuzumab (GA101) plus CHOP or FC in relapsed/refractory follicular lymphoma: Results of the GAUDI study (BO21000). Blood.

[18] Herrmann A., Hoster E., Zwingers T., Brittinger G., Engelhard M., Meusers P., Reiser M., Forstpointner R., Metzner B., Peter N. (2009). Improvement of Overall Survival in Advanced Stage Mantle Cell Lymphoma. J. Clin. Oncol..

[19] Hoster E., Dreyling M., Klapper W., Gisselbrecht C., Van Hoof A., Kluin-Nelemans J.C., Pfreundschuh M., Reiser M., Metzner B., Einsele H. (2008). A new prognostic index (MIPI) for patients with advanced-stage mantle cell lymphoma. Blood.

[20] Tiemann M., Schrader C., Klapper W., Dreyling M.H., Campo E., Norton A., Berger F., Kluin P., Ott G., Pileri S. (2005). Histopathology, cell proliferation indices and clinical outcome in 304 patients with mantle cell lymphoma (MCL): A clinicopathological study from the European MCL Network. Br. J. Haematol..

[21] Majlis A., Pugh W.C., Rodriguez M.A., Benedict W.F., Cabanillas F. (1997). Mantle cell lymphoma: Correlation of clinical outcome and biologic features with three histologic variants. J. Clin. Oncol..

[22] Ferrero S., Rossi D., Rinaldi A., Bruscaggin A., Spina V., Eskelund C.W., Evangelista A., Moia R., Kwee I., Dahl C. (2019). KMT2D mutations and TP53 disruptions are poor prognostic biomarkers in mantle cell lymphoma receiving high-dose therapy: A FIL study. Haematologica.

[23] Rummel M.J., Niederle N., Maschmeyer G., Banat G.A., von Grünhagen U., Losem C., Kofahl-Krause D., Heil G., Welslau M., Balser C. (2013). Bendamustine plus rituximab versus CHOP plus rituximab as first-line treatment for patients with indolent and mantle-cell lymphomas: An open-label, multicentre, randomised, phase 3 non-inferiority trial. Lancet.

[24] Flinn I.W., Van Der Jagt R., Kahl B., Wood P., Hawkins T., Macdonald D., Simpson D., Kolibaba K., Issa S., Chang J. (2019). First-Line Treatment of Patients with Indolent Non-Hodgkin Lymphoma or Mantle-Cell Lymphoma With Bendamustine Plus Rituximab Versus R-CHOP or R-CVP: Results of the BRIGHT 5-Year Follow-Up Study. J. Clin. Oncol..

[25] Romaguera J.E., Fayad L., Rodriguez M.A., Broglio K.R., Hagemeister F.B., Pro B., McLaughlin P., Younes A., Samaniego F., Goy A. (2005). High Rate of Durable Remissions After Treatment of Newly Diagnosed Aggressive Mantle-Cell Lymphoma with Rituximab Plus Hyper-CVAD Alternating with Rituximab Plus High-Dose Methotrexate and Cytarabine. J. Clin. Oncol..

[26] The Nobel Prize in Physiology or Medicine 1908. https://www.nobelprize.org/prizes/medicine/1908/summary/.

[27] Allison J., McIntyre B.W., Bloch D. (1982). Tumor-specific antigen of murine T-lymphoma defined with monoclonal antibody. J. Immunol..

[28] Kuhns M.S., Badgandi H.B. (2012). Piecing together the family portrait of TCR-CD3 complexes. Immunol. Rev..

[29] Rosenberg S.A., Packard B.S., Aebersold P.M., Solomon D., Topalian S.L., Toy S.T., Simon P., Lotze M.T., Yang J.C.-H., Seipp C.A. (1988). Use of Tumor-Infiltrating Lymphocytes and Interleukin-2 in the Immunotherapy of Patients with Metastatic Melanoma. N. Engl. J. Med..

[30] Rosenberg S.A., Aebersold P., Cornetta K., Kasid A., Morgan R.A., Moen R., Karson E.M., Lotze M.T., Yang J.C., Topalian S.L. (1990). Gene Transfer into Humans — Immunotherapy of Patients with Advanced Melanoma, Using Tumor-Infiltrating Lymphocytes Modified by Retroviral Gene Transduction. N. Engl. J. Med..

[31] Kuwana Y., Asakura Y., Utsunomiya N., Nakanishi M., Arata Y., Itoh S., Nagase F., Kurosawa Y. (1987). Expression of chimeric receptor composed of immunoglobulin-derived V regions and T-cell receptor-derived C regions. Biochem. Biophys. Res. Commun..

[32] Gross G., Waks T., Eshhar Z. (1989). Expression of immunoglobulin-T-cell receptor chimeric molecules as functional receptors with antibody-type specificity. Proc. Natl. Acad. Sci. USA.

[33] Neelapu S.S., Tummala S., Kebriaei P., Wierda W., Gutierrez C., Locke F.L., Komanduri K.V., Lin Y., Jain N., Daver N. (2018). Chimeric antigen receptor T-cell therapy — assessment and management of toxicities. Nat. Rev. Clin. Oncol..

[34] Lee D.W., Gardner R., Porter D.L., Louis C.U., Ahmed N., Jensen M., Grupp S.A., Mackall C.L. (2014). Current concepts in the diagnosis and management of cytokine release syndrome. Blood.

[35] Gust J., Hay K.A., Hanafi L.-A., Li D., Myerson D., Gonzalez-Cuyar L.F., Yeung C., Liles W.C., Wurfel M., Lopez J.A. (2017). Endothelial Activation and Blood–Brain Barrier Disruption in Neurotoxicity after Adoptive Immunotherapy with CD19 CAR-T Cells. Cancer Discov..

[36] Locke F.L., Ghobadi A., Jacobson C.A., Miklos D.B., Lekakis L.J., Oluwole O., Lin Y., Braunschweig I., Hill B.T., Timmerman J.M. (2019). Long-term safety and activity of axicabtagene ciloleucel in refractory large B-cell lymphoma (ZUMA-1): A single-arm, multicentre, phase 1–2 trial. Lancet Oncol..

[37] Nastoupil L.J., Jain M., Feng L., Spiegel J.Y., Ghobadi A., Lin Y., Dahiya S., Lunning M., Lekakis L., Reagan P. (2020). Standard-of-Care Axicabtagene Ciloleucel for Relapsed or Refractory Large B-Cell Lymphoma: Results from the US Lymphoma CAR T Consortium. J. Clin. Oncol..

[38] Jacobson M.C., Chavez J.C., Sehgal A.R., William M.B.M., Munoz J., Salles M.G., Munshi P.N., Casulo C., Maloney D., de Vos S. (2020). Primary Analysis of Zuma-5: A Phase 2 Study of Axicabtagene Ciloleucel (Axi-Cel) in Patients with Relapsed/Refractory (R/R) Indolent Non-Hodgkin Lymphoma (iNHL). Blood.

[39] Jaeger U. (2020). Myc Expression and Tumor-Infiltrating T Cells Are Associated with Response in Patients (Pts) with Relapsed/Refractory Diffuse Large B-Cell Lymphoma (r/r DLBCL) Treated with Tisagenlecleucel in the Juliet Trial. Proceedings of the 62nd ASH Annual Meeting and Exposition.

[40] Chong E.A., Ruella M., Schuster S.J. (2021). Five-Year Outcomes for Refractory B-Cell Lymphomas with CAR T-Cell Therapy. N. Engl. J. Med..

[41] Pasquini M.C., Hu Z.-H., Curran K., Laetsch T., Locke F., Rouce R., Pulsipher M.A., Phillips C.L., Keating A., Frigault M.J. (2020). Real-world evidence of tisagenlecleucel for pediatric acute lymphoblastic leukemia and non-Hodgkin lymphoma. Blood Adv..

[42] Upadhaya S., Yu J.X., Shah M., Correa D., Partridge T., Campbell J. (2021). The clinical pipeline for cancer cell therapies. Nat. Rev. Drug Discov..

[43] Yang S., Li J.-Y., Xu W. (2014). Role of BAFF/BAFF-R axis in B-cell non-Hodgkin lymphoma. Crit. Rev. Oncol..

[44] Qin H., Dong Z., Wang X., Cheng W.A., Wen F., Xue W., Sun H., Walter M., Wei G., Smith D.L. (2019). CAR T cells targeting BAFF-R can overcome CD19 antigen loss in B cell malignancies. Sci. Transl. Med..

[45] Ormhøj M., Scarfò I., Cabral M.L., Bailey S., Lorrey S.J., Bouffard A.A., Castano A.P., Larson R.C., Riley L.S., Schmidts A. (2019). Chimeric Antigen Receptor T Cells Targeting CD79b Show Efficacy in Lymphoma with or without Cotargeting CD19. Clin. Cancer Res..

[46] Ramos C.A., Savoldo B., Torrano V., Ballard B., Zhang H., Dakhova O., Liu E., Carrum G., Kamble R.T., Gee A.P. (2016). Clinical responses with T lymphocytes targeting malignancy-associated κ light chains. J. Clin. Investig..

[47] Köksal H., Dillard P., Josefsson S.E., Maggadottir S.M., Pollmann S., Fåne A., Blaker Y.N., Beiske K., Huse K., Kolstad A. (2019). Preclinical development of CD37CAR T-cell therapy for treatment of B-cell lymphoma. Blood Adv..

[48] Dai H., Wu Z., Jia H., Tong C., Guo Y., Ti D., Han X., Liu Y., Zhang W., Wang C. (2020). Bispecific CAR-T cells targeting both CD19 and CD22 for therapy of adults with relapsed or refractory B cell acute lymphoblastic leukemia. J. Hematol. Oncol..

[49] Ghafouri S.N., Walthers C., Roshandell M., Ji B., Trent J., Chen J.M., Naparstek J., Harris C., Schweppe T., Nawaly K.K. (2021). Abstract CT007: CD19/CD20 Bispecific Chimeric Antigen Receptor (CAR) in Naive/Memory T-Cells for the Treatment of Relapsed or Refractory B-Cell Lymphomas. Clinical Trials.

[50] Han X., Wang Y., Wei J., Han W. (2019). Multi-antigen-targeted chimeric antigen receptor T cells for cancer therapy. J. Hematol. Oncol..

[51] Hossain N., Sahaf B., Abramian M., Spiegel J.Y., Kong K., Kim S., Mavroukakis S., Oak J., Natkunam Y., Meyer E.H. (2018). Phase I Experience with a Bi-Specific CAR Targeting CD19 and CD22 in Adults with B-Cell Malignancies. Blood.

[52] Cronk R.J., Zurko J., Shah N.N. (2020). Bispecific Chimeric Antigen Receptor T Cell Therapy for B Cell Malignancies and Multiple Myeloma. Cancers.

[53] Torikai H., Reik A., Soldner F., Warren E., Yuen C., Zhou Y., Crossland D.L., Huls H., Littman N., Zhang Z. (2013). Toward eliminating HLA class I expression to generate universal cells from allogeneic donors. Blood.

[54] Poirot L., Philip B., Schiffer-Mannioui C., Le Clerre D., Chion-Sotinel I., Derniame S., Potrel P., Bas C., Lemaire L., Galetto R. (2015). Multiplex Genome-Edited T-cell Manufacturing Platform for “Off-the-Shelf” Adoptive T-cell Immunotherapies. Cancer Res..

[55] Georgiadis C., Preece R., Nickolay L., Etuk A., Petrova A., Ladon D., Danyi A., Humphryes-Kirilov N., Ajetunmobi A., Kim D. (2018). Long Terminal Repeat CRISPR-CAR-Coupled “Universal” T Cells Mediate Potent Anti-leukemic Effects. Mol. Ther..

[56] DiPersio J.F., Staser K., Cooper M. (2020). Immunotherapy for T-Cell ALL and T-Cell NHL. Clin. Lymphoma Myeloma Leuk..

[57] Depil S., Duchateau P., Grupp S.A., Mufti G., Poirot L. (2020). ‘Off-the-shelf’ allogeneic CAR T cells: Development and challenges. Nat. Rev. Drug Discov..

[58] Cooper M.L., Choi J., Staser K., Ritchey J.K., Devenport J.M., Eckardt K., Rettig M.P., Wang B., Eissenberg L.G., Ghobadi A. (2018). An “off-the-shelf” fratricide-resistant CAR-T for the treatment of T cell hematologic malignancies. Leukemia.

[59] Perez C., Gruber I., Arber C. (2020). Off-the-Shelf Allogeneic T Cell Therapies for Cancer: Opportunities and Challenges Using Naturally Occurring “Universal” Donor T Cells. Front. Immunol..

[60] Ayyappan S., Maddocks K. (2019). Novel and emerging therapies for B cell lymphoma. J. Hematol. Oncol..

[61] Huang R., Li X., He Y., Zhu W., Gao L., Liu Y., Wen Q., Zhong J.F., Zhang C., Zhang X. (2020). Recent advances in CAR-T cell engineering. J. Hematol. Oncol..

[62] Chmielewski M., Abken H. (2015). TRUCKs: The fourth generation of CARs. Expert Opin. Biol. Ther..

[63] Qin L., Zhao R., Chen D., Wei X., Wu Q., Long Y., Jiang Z., Li Y., Wu H., Zhang X. (2020). Chimeric antigen receptor T cells targeting PD-L1 suppress tumor growth. Biomark. Res..

[64] Chong E.A., Melenhorst J.J., Lacey S.F., Ambrose D.E., Gonzalez V., Levine B.L., June C.H., Schuster S.J. (2017). PD-1 blockade modulates chimeric antigen receptor (CAR)–modified T cells: Refueling the CAR. Blood.

[65] Chong E.A., Svoboda J., Nasta S.D., Landsburg D.J., Winchell N., Napier E., Mato A.R., Melenhorst J.J., Ruella M., Lacey S.F. (2018). Sequential Anti-CD19 Directed Chimeric Antigen Receptor Modified T-Cell Therapy (CART19) and PD-1 Blockade with Pembrolizumab in Patients with Relapsed or Refractory B-Cell Non-Hodgkin Lymphomas. Blood.

[66] Jacobson C.A., Locke F.L., Miklos D.B., Herrera A.F., Westin J.R., Lee J., Rossi J.M., Zheng L., Avanzi M.P., Roberts Z.J. (2018). End of Phase 1 Results from Zuma-6: Axicabtagene Ciloleucel (Axi-Cel) in Combination with Atezolizumab for the Treatment of Patients with Refractory Diffuse Large B Cell Lymphoma. Blood.

[67] Lee D.W., Santomasso B.D., Locke F.L., Ghobadi A., Turtle C.J., Brudno J.N., Maus M.V., Park J.H., Mead E., Pavletic S. (2019). ASTCT Consensus Grading for Cytokine Release Syndrome and Neurologic Toxicity Associated with Immune Effector Cells. Biol. Blood Marrow Transplant..

[68] Ying Z., Huang X.F., Xiang X., Liu Y., Kang X., Song Y., Guo X., Liu H., Ding N., Zhang T. (2019). A safe and potent anti-CD19 CAR T cell therapy. Nat. Med..

[69] Crunkhorn S. (2021). Switching CAR-T cells on and off. Nat. Rev. Drug Discov..

